# Mid-term to long-term results of primary arthroscopic Bankart repair for traumatic anterior shoulder instability: a retrospective study

**DOI:** 10.1186/s12891-020-03223-3

**Published:** 2020-03-27

**Authors:** Benjamin Panzram, Yasser Kentar, Michael Maier, Thomas Bruckner, Pit Hetto, Felix Zeifang

**Affiliations:** 1 Department of Orthopaedic Surgery, Trauma Surgery and Spinal Cord Injury, University of Heidelberg, Heidelberg, Germany; 2 Heidelberg University Hospital, Clinic for Orthopaedics and Trauma Surgery, Schlierbacher Landstraße 200A, 69118 Heidelberg, Germany; 3 Institute of Medical Biometry and Informatics, University of Heidelberg, Heidelberg, Germany; 4 Ethianum Clinic Heidelberg, Heidelberg, Germany

**Keywords:** Shoulder, Instability, Arthroscopic, Bankart repair, Suture anchors

## Abstract

**Background:**

The arthroscopic method offers a less invasive technique of Bankart repair for traumatic anterior shoulder instability. The aim of the study is to determine the mid−/long-term functional outcome, failure rates and predictors of failure after primary arthroscopic Bankart repair for traumatic anterior shoulder instability.

**Methods:**

A total of 100 patients were primarily operated using arthroscopic Bankart repair after traumatic anterior shoulder instability. Medical records were retrospectively reviewed, and patients were assessed using postal questionnaire after a mean follow-up of 8.3 years [3–14]. Clinical assessment was performed using Constant score, Rowe score, and American Shoulder and Elbow Surgeons score.

**Results:**

The overall recurrence rate was 22%. The Kaplan-Meier failure-free survival estimates.

were 80% at 5 years and 70% at 10 years. Nearly half (54.5%) of recurrences occurred at 2 years postoperative. Compared with normal shoulder, there were statistical differences in all 3 scores. Failure rate was significantly affected by age at the time of surgery with 86% of recurrence cases observed in patients aged 30 years or younger. Nevertheless, Younger age at the time of surgery (*P* = 0.007) as well age at the time of initial instability (*P* = 0.03) was found to correlate negatively with early recurrence within 2 years of surgery. Among those with recurrent instability, recurrence rate was found to be higher if there had been more than 5 instability episodes preoperatively (*P* = 0.01). Return to the preinjury sport and occupational level was possible in 41 and 78%, respectively.

**Conclusion:**

Failure-free survival rates dropped dramatically over time. Alternative reconstruction techniques should be considered in those aged ≤30 years due to the high recurrence rate.

## Background

Various open and arthroscopic techniques have been developed overtime to address the glenohumeral joint instability with the ultimate goal of restoring the shoulder function and lowering the rate of recurrent instability over the long-term. Bankart repair is currently the treatment of choice according to various surveys of surgeons, with > 90% of surgeons choosing the Bankart procedure as initial repair procedure [3, 4, 14]. A review of the American Board of Orthopaedic Surgery revealed a significant increase in the percentage of arthroscopically-performed Bankart repairs was noted, being performed in approximately 88% of cases between 2006 and 2008, compared to 71% in the period from 2003 to 2005 [35]. In Germany, more than 90% use arthroscopic techniques in the first-time dislocation and more than 70% even in chronic instability, as long as there is not significant bone loss [3]. One of the major advantages of the arthroscopic surgery is the precise identification of the intra-articular pathology with minor soft tissue dissection. In the period between 1995 through 2004, the arthroscopic approach using metallic staples, tacks and transglenoid suturing had twice the risk of postoperative recurrence of instability compared with the open repair [25]. Given the rapid evolution of arthroscopic equipments, techniques, and surgeon’s experience, several recent studies showed no superiority of the open technique over the arthroscopic Bankart repair using suture anchors [7, 16, 22, 36].

In respect to recurrent instability after the arthroscopic Bankart repair using suture anchors, the rates have varied widely in the available literature ranging between 3 and 41% [1, 11, 15, 17, 18, 24, 26, 50, 53]. Several contributors such as the sample size, length of follow up and the definition of postoperative failure might explain this heterogeneity in the aforementioned studies. Several factors including significant bone defects, young age, the number of previous dislocations, time to surgery as well as participation in contact sports could be associated with an increased risk of recurrent instability [2, 8, 37, 38].

The purpose of this study was to report on our experience with arthroscopic Bankart repair using suture anchors and to determine, through a retrospective case series, factors potentially associated with increased postoperative recurrence of instability.

## Methods

### Patient selection

We retrospectively reviewed a consecutive series of patients who underwent primary arthroscopic Bankart repair using suture anchors in the period between January 2004 and December 2014. All patients had a history of a traumatic injury to their shoulder resulting in an anteroinferior shoulder instability confirmed on history, physical examination and magnetic resonance imaging findings. Exclusion criteria were (1) extension of the labrum lesion into the superior labrum (I.e., superior labrum anterior-posterior tear) or posteriorly, (2) atraumatic or multidirectional instability or posterior instability, (3) concomitant rotator cuff tears, (4) arthroscopic stabilization after failed previous repair. A written consent was obtained by all patients who participated in our study.

A total of 100 shoulders fulfilled the study inclusion criteria and were available at the time of the final review.

### Operative technique

Operative technique was carried out in a Beach-chair position using a standard posterior viewing portal in addition to two anterior working portals in the rotator interval, as described in several publications [23, 39, 46].

After a standardized diagnostic arthroscopy, the Bankart lesion was confirmed and evaluated. Using a Bankart-Chisel, the detached labrum, was mobilized and elevated from the anterior glenoid. An arthroscopic rasp or a shaver was used to create a bleeding bed along the glenoid edge. In all patients, a capsular plication was performed in addition. Using a suture passing instrument, a suture was advanced through the capsulolabral complex to be used as a shuttle suture. Drill holes were created on the glenoid at the 3 and 5:30 o’clock position for the right shoulder and the 6:30 and 9 o’clock for the left shoulder. Additional anchors were placed as necessary. According to the extent of the capsulolabral defect, one to four anchors were used: PushLock® (Arthrex), PANALOK® (DePuy) and Lupine® Loop (DePuy).

### Postoperative management

A sling immobilizer was applied after surgery and worn for 2 weeks. Physical therapy was initiated in the first day after surgery with passive exercises for flexion/abduction/external rotation 60°-60°-0°. Active-assisted range of motion was started after 1 week with gradually increase. No heavy lifting and carrying loads over 5 kg and no contact sports for 12 weeks after operation.

### Clinical evaluation and outcome measurement

We extracted preoperative and intraoperative data retrospectively from medical records. Preoperative assessment included age, age at surgery, age at time of injury, time to surgery (interval between the first instability event and surgery) and number of instability events prior to repair. Participants were divided into 2 groups: Group A with only one episode of subluxation/dislocation and group B with more than one episode. Group B was further subdivided into subgroup B1 with 2–5 episodes and subgroup B2 with more than 5 episodes. Intraoperative findings included type and number of anchors used in the repair. A structured questionnaire was mailed and asked to provide type and level of sport activity as well as profession prior to injury. In respect to the type of sport activity prior to injury, participants were grouped in 3 categories: (1) non-contact sports such as tennis, golf, swimming and running, (2) limited-contact sports such as basketball and soccer (3) full-contact sports: such as football, boxing and rugby. According to the level of sport activity, participants were divided intro 3 categories: (1) non-athletes, (2) recreational athletes, and (3) competitive athletes. Type of profession was also classified in 3 groups: group (1) characterizes professions associated with low loads on the shoulder such as office workers, teachers and students, group (2) characterizes professions with moderate loads on the shoulder such as nurses, cooks and gardeners, and group (3) characterizes professions with high loads on the shoulder such as roofers, electrical workers and painters. Postoperative assessment included recurrence of instability where only physician-documented instabilities were considered as a treatment failure. Furthermore, early postoperative recurrence was defined in the current study as those occurring within 2 years of surgery. Clinical outcome and functional activity levels were evaluated using the American Shoulder and Elbow Surgeons Score (ASES), the self-assessment score of shoulder function based on Rowe score [29], and Constant-Murley score. In respect to the strength measurement in the Constant-Murley score, the participants were asked to lift an object with known mass (e.g., water bottle 1 Liter) and hold it for 5 s in abduction 90° and slight flexion 20°. One point is given per 0.5 kg. Postoperatively, the level of sport activity as well as the level of occupational performance was measured at six-point licker scale as follows: 1) no change; 2) minimal decrease; 3) slight decrease; 4) moderate decrease; 5) severe decrease; and 6) very severe decrease.

### Statistical analysis

Statistical analysis was performed using SPSS software Version 22 for Windows (SPSS Inc.). Normal distribution of data was tested using the Kolmogorov-Smirnov test before using the Student t test for parametric data or the Mann-Whitney U test for nonparametric data. The chi-square test was used to assess differences between categorical data. In respect to the clinical outcome scores, differences were analyzed by 2-tailed, paired t-test. The level of significance was set at a *p* value of < 0.05. Pearson and Spearman correlation tests were used to detect the statistical significance of age/age intervals. The distribution of continuous data such as age, time to surgery and number of anchors used was provided in the form of means, standard deviation, and interquartile range 25–75%. Scores results were given in the form of means and standard deviation. Categorical variables were illustrated by absolute and relative frequencies (count and percentage).

## Results

Base characteristics of the study cohort are presented in (Table 1).

**Table 1:** Baseline characteristics of patients who underwent arthroscopic Bankart repair (*n* = 100)

Variable	Data*
Age (years)	37(29–33)
Age at surgery (years)	27.8(19–25)
Age at first instability event (years)	23.6(17–29)
Gender	
Male	76(76%)
Female	34(24%)
Time to surgery (months)	51.4(2–57)
Preoperative instability episodes	
Group A: 1	33(33%)
Group B: > 1	67(67%)
Group B1 [2–5]	27(40.2%)
Group B2 (> 5)	40(59.8%)
Dominant side affected	
Yes	57(57%)
No	43(43%)
No. of suture anchors used in Bankart repair	2.5(2–3)
Preoperative type of sport activity	
Non-contact sports	43(43%)
Limited-contact sports	25(25%)
Full-contact sports	32(32%)
Preoperative level of sport	
Non-athletes	28(28%)
Recreational-athletes	39(39%)
Competitive-athletes	33(33%)
Level of occupational shoulder stress	
Low stress	69(69%)
Moderate stress	11(11%)
High stress	20(20%)

*Values are mean, interquartile range (25–75%) or n (%)

The mean follow-up was 8.3 years (range, 3–14 years). Results of Constant-Murley Score, modified Rowe Score, and American Shoulder and Elbow Surgeons (ASES) Score for both affected/unaffected sides are presented in (Table 2). All scores were postoperative significantly lower than measured values on the unaffected arm. Changes in level of sport activity as well as occupational performance are presented in (Table 3). Overall, 41% of participants (41 patients) were able to keep the same preoperative sport level, whereas 78% (78 patients) returned to previous occupational level.

**Table 2:** Clinical outcomes after arthroscopic Bankart repair (*n* = 100)

Outcome measure	Affected arm**	Unaffected arm	***P value
Constant-Murley score	87 ± 16	93.4 ± 8.9	0.000
Modified Rowe score	91.9 ± 62.9	95.8 ± 8.5	0.000
ASES score*	87.4 ± 16.3	96.7 ± 8.2	0.000

*American Shoulder and Elbow Surgeons score, ** Values are mean ± standard deviation (SD), *** Significance at *P* < 0.05

**Table 3:** Level of sport and occupational performance decreases after arthroscopic repair

6-point Likert scale	Sport performance*	Occupational performance
Normal	41 (41%)	78 (78%)
Minimal	21 (21%)	11 (11%)
Slight	14 (14%)	8 (8%)
Moderate	9 (9%)	1 (1%)
Severe	11 (11%)	2 (2%)
Very severe	4 (4%)	0

*Values are n (%)

Among the 100 shoulders with a follow-up of at least 3 years, postoperative recurrence of instability was reported in 22% of cases (22 patients). Four participants reported only one episode of postoperative instability; 2 treated conservatively and 2 surgically. On the other hand, 18 participants (18%) reported two or more instability events; 11 treated conservatively and 7 surgically. The following parameters were found to have a significant negative correlation with postoperative recurrence instability:

### Age

**Fig. 1:**
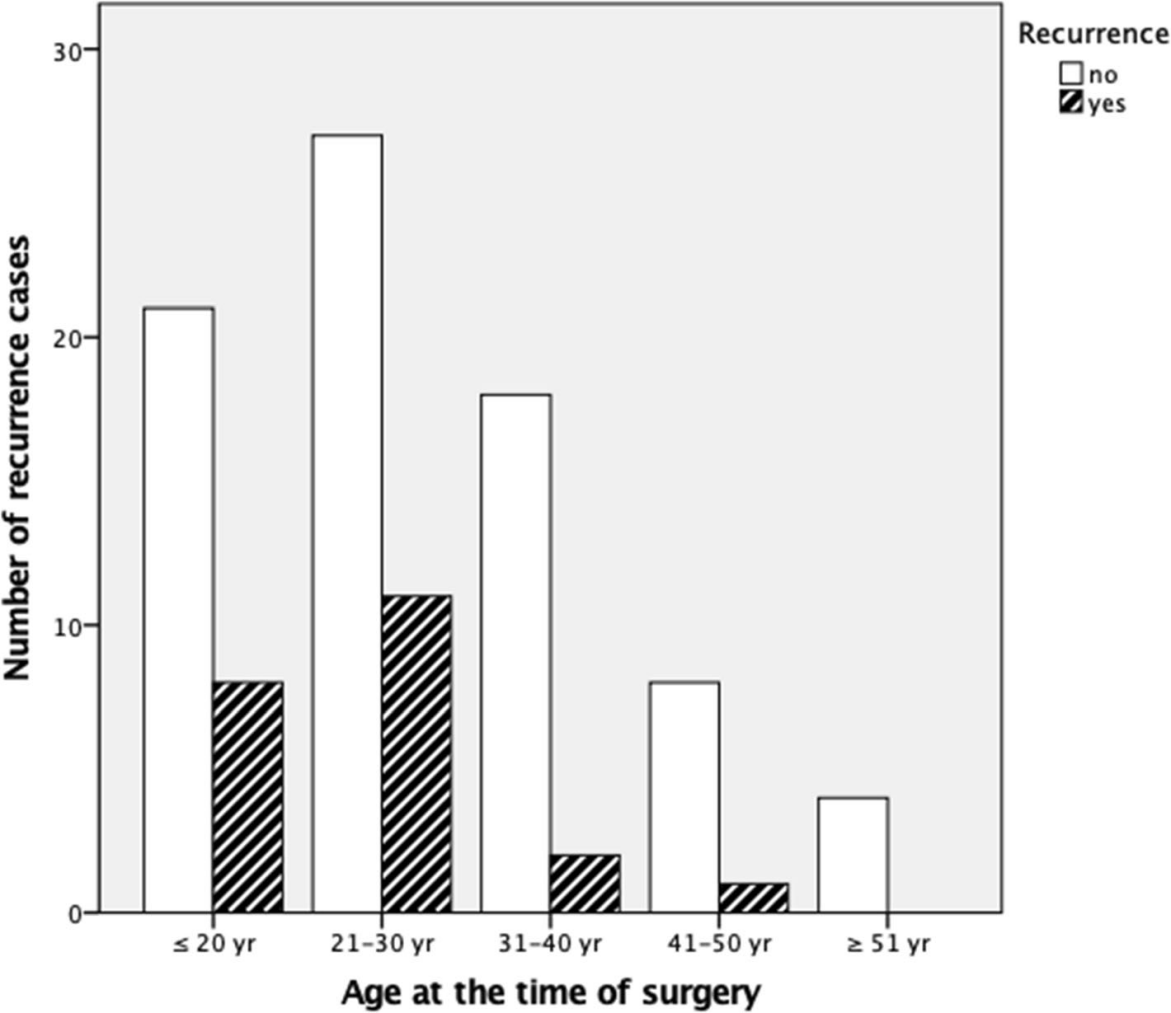
Recurrence rates by age at the time of surgery at 10-year intervals

At the time of data analysis, participants who reported recurrence were on average 4 years younger than those who did not (*p* = 0.11). There was a statistically significant negative correlation between younger age at the time of surgery and recurrence rate (*p* = 0.019, Pearson correlation coefficient = 0.01). Of the 22 recurrence cases, 86% of participants were ≤ 30 years old at the time of surgery (*p* = 0.029, Spearman correlation coefficient = 0.009). On the other hand, age at time of initial instability was not found to be statistically significant in respect to the recurrence rate (*p* = 0.14, Pearson correlation coefficient = 0.12). Recurrence rates by age at the time of surgery at 10-year intervals are shown in (Fig. 1).

### Number of preoperative instability episodes

In group A which includes patients who sustained only one episode of subluxation/dislocation prior surgery, a recurrence rate of 21% was observed. This was statistically not significant (*p* = 0.89) compared to group B (22% recurrence rate) which includes patients with more than one episode. However, a statistically significant difference (*p* = 0.003) was found between both subgroups B1, which included those who sustained 2–5 preoperative episodes of instability (3.7% recurrence rate) and B2, which included those with more than 5 episodes (35% recurrence rate).

Other factors including gender, time to surgery, dominant side, number of suture anchors used as well as preoperative sport level and occupational shoulder stress were not found to have a significant association with postoperative recurrence of instability. Table 4 summarizes the results observed in both groups.

**Table 4:** Associated factors with postoperative recurrence of instability after arthroscopic Bankart repair (*n* = 100)

Variable	Recurrence*		*P* value**
	Yes (*n* = 22)	No (*n* = 88)	
Age (years)	33.7 ± 9.3	37.8 ± 10.6	0.11
Age at surgery (years)	24 ± 7.7	28.9 ± 10.9	**0.019**
Age at first instability event (years)	21.4 ± 8.5	24.2 ± 9.9	0.14
Gender			
Male	17 (22.4%)	59 (77.6%)	0.87
Female	5 (20.8%)	19 (79.2%)	
Time to surgery (months)	31.9 ± 47.2	56.8 ± 91.9	0.73
Preoperative instability episodes			
Group A: 1	7 (21.2%)	26 (78.8%)	0.89
Group B: > 1	15 (22.4%)	52 (77.6%)	
Group B1 (2–5)	1 (3.7%)	26 (96.3%)	**0.003**
Group B2 (> 5)	14 (35%)	26 (65%)	
Dominant side affected			0.45
Yes	11 (9.3%)	46 (80.7%)	
No	11 (25.6%)	32 (74.7%)	
No. of suture anchors used in Bankart repair	2.4 ± 0.6	2.4 ± 0.6	0.88
Anchor type			0.34
PushLock®	8 (32%)	17 (68%)	
PANALOK®	4 (22.2%)	14 (77.8%)	
LUPINE®	10 (17.5%)	47 (82.5%)	
Preoperative type of sport activity			0.45
Non-contact sports	7 (16.3%)	36 (83.7%)	
Limited-contact sports	6 (24%)	19 (76%)	
Full-contact sports	9 (28.1%)	23 (71.9%)	
Level of occupational shoulder stress			0.086
Low stress	19 (27.5%)	50 (72.5%)	
Moderate stress	0	11 (100%)	
High stress	3 (15%)	17 (85%)	

*Values are mean± standard deviation (SD) or n (%). ** Significance at *P*< 0.05

#### Recurrence rates of arthroscopic Bankart repair using Kaplan-Meier analysis

**Fig. 2:**
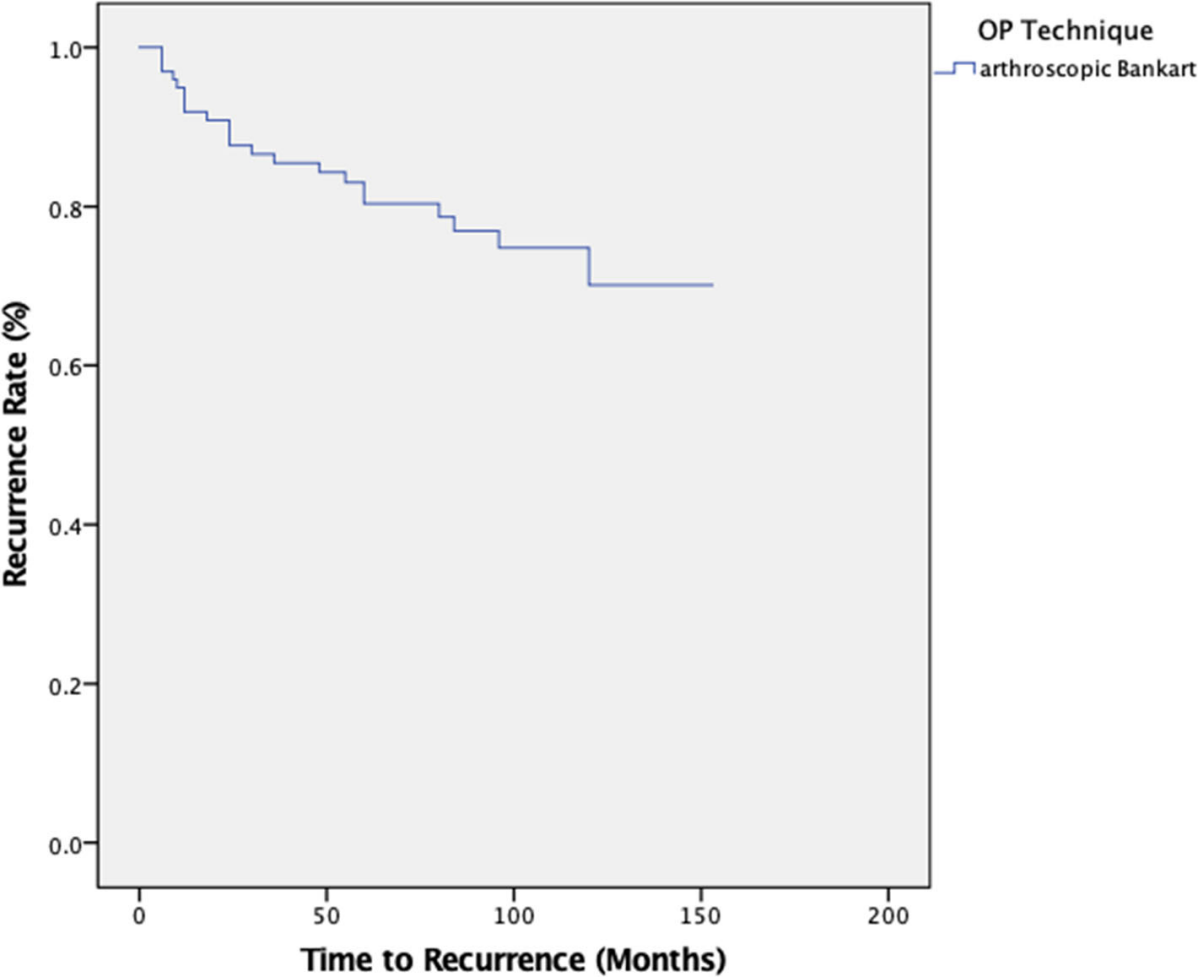
Recurrence-free survival estimates for arthroscopic Bankart repair

The Kaplan-Meier survival curve for recurrence-free survival is shown in (Fig. 2). The mean estimate for the cumulative proportion of stable shoulders at a mean follow-up of 8 years was 123 months (SD = 5.6, 95% confidence interval (112–134)). The recurrence-free survival estimates at 1 year, 2 years, 5 years, and 10 years were 91, 87, 80, and 70%, respectively.

#### Early versus late postoperative recurrence

Twelve patients who developed recurrence within 24 months after surgery were defined as having early recurrence (54.5%). In this group, 4 patients suffered a trauma-based postoperative instability: 1 patient experienced following a fall while playing football and 3 patients suffered traumatic recurrence due to physical trauma unrelated to sport. The other 8 recurrence cases occurred during performing tasks of daily activities: 5 while reaching for overhead objects, 2 while sleeping and one by stretching exercises. Seven patients were treated conservatively and 5 operatively.

On the other Hand, 10 patients developed recurrence after 24 months of surgery and were defined as having late recurrence (55.5%). Six patients suffered postoperative instability following a non-traumatic event: 4 while reaching for overhead objects and 2 while sleeping. Traumatic events have been reported in the other 4 cases: 1 following a motorcycle collision, 2 after a fall while descending stairs and 1 sustained through skiing. Six patients were treated conservatively and 4 required additional surgery to restore instability.

Both age at the time of surgery as well as age at the time of initial instability showed a significant negative correlation with postoperative recurrence within 2 years. Table 5 summarizes the main differences of patients with early versus late recurrence following Bankart repair for anterior shoulder instability.

**Table 5:** Characteristics of patients with early versus late recurrence following Bankart repair for traumatic anterior shoulder instability

Variable	Recurrence*		*P* value**
	Early (*n* = 12)	Late (*n* = 10)	
Age (years)	29.67 ± 5.4	40.7 ± 14.4	**0.004**
Age at surgery (years)	21.85 ± 5.1	31.6 ± 12.4	**0.007**
Age at first instability event (years)	19.1 ± 6.2	27.1 ± 12.6	**0.03**
Gender			
Male	9 (75%)	7 (70%)	0.79
Female	3 (25%)	3 (30%)	
Mechanism of recurrence			
Traumatic	4 (33.3%)	4 (40%)	0.74
Non-traumatic	8 (66.7%)	6 (60%)	
Time to surgery (months)	27.3 ± 38.4	55.1 ± 65.5	0.093
Preoperative instability episodes			
Group A: 1	2 (16.7%)	4 (40%)	0.22
Group B: > 1	10 (83.3%)	6 (60%)	
Group B1 [2–5]	1 (10%)	2 (33.3%)	0.24
Group B2 (> 5)	9 (90%)	4 (66.6%)	
Dominant side affected			0.39
Yes	7 (58.3%)	4 (40%)	
No	5 (41.7%)	6 (60%)	
No. of suture anchors used in Bankart repair	2.5 ± 0.6	2.3 ± 0.6	0.52
Preoperative type of sport activity			
Non-contact sports	5 (41.7%)	3 (30%)	
Limited-contact sports	3 (25%)	3 (30%)	
Full-contact sports	4 (33.3%)	4 (40%)	0.85
Level of occupational shoulder stress			
Low stress	11 (91.7%)	9 (90%)	
Moderate stress	0	0	
High stress	1 (8.3%)	1 (10%)	0.089

* Values are mean ± standard deviation (SD) or n (%). ** Significance at *P* < 0.05

## Discussion

The current study included 100 patients who underwent arthroscopic Bankart repair with a mean follow-up of 8.3 years. The postoperative failure rate was 22%. In comparison with the available literature, the findings of the present study appear to be consistent with that of previous studies with long-term follow-up [1, 11, 12, 18]. Castagna et al. reported a recurrence rate at 22.5% at mean follow-up of 10.9 years [11]. Franceschi et al. reported a recurrence rate at 17% at mean follow-up of 8 years [18]. Similarly, at minimum follow-up of 13 years, recurrence rate was found to be approximately 18% [1]. Higher rates of recurrence were reported by Zimmermann at mean follow-up of 6 years (41%), Van der Linde et al. after a mean follow-up of 9 years (35%), and recently by Flinkkila et al. at minimum follow-up of 10 years (30%) [17, 50, 53]. The higher recurrence rate in these studies compared to the current investigation could be explained by the fact that the authors defined postoperative failure as any subjective feeling of instability regardless the findings of physical and radiological examinations. They argued that including only patients with physician-documented dislocations/subluxations represents an underestimation of the effect of surgical therapy. Other investigations with smaller sample sizes and/or shorter follow-up periods (24–40 months) reported remarkably lower recurrence rates (range, 3.4–11%) [13, 15, 21, 24, 26, 32, 34].

In the current study, recurrence rate appeared to drop significantly with advanced age (*p* = 0.019). This has been supported in several investigations [27, 30, 34, 38, 40, 49]. Eighty-six percent of the observed recurrence cases in the current study occurred in patients with the age ≤ 30 years at the time of surgery (*p* = 0.029). Flinkkila et al. reported in a retrospective study with a minimum of 10 years follow up a remarkably higher recurrence rate in patients younger than 20 years old at the time of surgery (54% vs. 24%, *p* < 0.001) [17]. This was also supported by Aboalata et al. in a retrospective study with a minimum follow-up of 13 years [1]. The authors divided their patients into 3 categories in respect to the age at surgery; group A included patients younger than 20 years, group B aged 21 to 30 years and group C older than 30 years. The recurrence rate was 39,1, 16.1% and 13,4%, respectively (*p* = 0.007). Considering a cut-off value of 22 years of age, two studies detected a negative correlation concerning age at the time of surgery and recurrence rate [27, 49]. On the contrary, other studies were not able to establish a significant cut off value [30, 34, 51]. It is worth mentioning that other studies showed that age at the time of first dislocation (the impact of age at the time of surgery was not taken into consideration) correlated negatively with instability [42–44, 48]. These studies discussed the risk factors for recurrence after anterior shoulder instability in general, not focusing on a specific surgical intervention. This could not be statistically supported in our study, which could be partly referred to the recall bias. Calvo et al. reported in a prospective study of 61 individuals who underwent arthroscopic Bankart repair that age at the time of surgery rather than the age at first dislocation, showed a negative correlation with postoperative failure [9]. Gender seems not to have an impact of postoperative recurrent instability after arthroscopic Bankart repair, which is supported in literature [38]. Furthermore, no association between the recurrence rate and side dominance could be detected, which is also consistent with previous research [2, 17, 38, 50].

Controversial results in respect to the impact of preoperative instability episodes have been presented in the literature. In the study by Imhoff et al., patients with a single preoperative dislocation had a significantly lower rate of postoperative recurrence than patients who had had more than one dislocation prior to arthroscopic repair [27]. A cut-off value of 5 episodes by Habermeyer et al. and less than 4 episodes by Jaeger et al. was reported [20, 28]. In the current investigation, recurrence rates appeared to be similar between those who sustained one episode vs. multiple episodes of instability prior to surgery (*p* = 0.89), which is consistent with several previous investigations [1, 2, 11, 38, 50]. However, recurrence rates among patients with more than 5 episodes were significantly higher (*p* = 0.003) than those among patients with 2 to 5 episodes.

The results of the current study indicate that the time interval between the first instability event and surgery has no influence on the postoperative recurrence rate (Mean = 57.8, *p* = 0.73). This is consistent with several previous reports [1, 15, 30, 51]. Porcellini et al. found a statistically significant proportional correlation as 68% of recurrence cases were observed in those who underwent surgery between 6 months and 12 months after initial dislocation in comparison to 32% in those who underwent surgery within 6 months (*p* = 0.01) [38]. They argued their findings by the capsule elongation and deformation which make the delayed reconstruction much more difficult.

The number of anchors used did not correlate significantly with the recurrence rate in the current study. Boileau et al. and Shibata et al. postulated that four anchors, at a minimum, should be used to secure a better stabilization regardless of the extent of the labral lesion. In both studies, patients who had 3 anchors or less showed significantly higher recurrence rate [6, 47]. On the contrary, several previous investigations could not show a significant correlation [1, 17, 33].

Return to the preinjury level of sport was possible in 40% of cases. However, this was not possible in 70% of patients who participated in full-contact sports. These results are in line with that recent result by Aboalata et al. [1]. Higher rates above 80% were reported by other authors [26, 32, 41]. No difference in terms of postoperative recurrence rate was observed in this study among the three sport groups (non-contact, limited-contact, and full-contact sports). Several publications reported increased risk of recurrence in contact-athletes [11, 52], whereas others did not find a significant difference [10, 26]. Unfortunately, there is a high variability in the classification of sport types that makes the comparison quite difficult. In the current study, approximately 78% of participants were able to return to the preinjury work level, in comparison to 97% reported by Franceschi and colleagues [18]. No significant differences of postoperative failure rates among the three work groups were observed.

All postoperative scores showed good (Constant-Murley Score: mean = 87 and ASES score: mean = 87.4) to excellent (modified Constant score: m = 94.2 and Rowe score: m = 91.9) results, although statistically lower than observed results on the non-affected arm. Similar findings were reported in the literature [1, 19, 31, 45]. Franceschi et al. reported a Constant score of 89 and Rowe score of 88 after a mean follow up of 8 years [18]. Excellent Constant, Rowe, and ASES scores (mean = 94, 90, 92, respectively) were reported by Aboalata et al. at minimum follow up of 10 years [1].

The current study showed that nearly half of recurrences occurred 2 years after primary surgery. After 2 years, Gerber et al. found that nearly 60% of recurrence cases (22/36 of total 271 patients-minimum follow-up 6 years) had not yet occurred [53]. At minimum follow-up of 10 years, Flinkkila et el. reported a similar trend with 50% of recurrence cases occurring after 2 years after surgery [17]. Likewise, Bessiere et al. and van der Linde et al. have provided further evidence regarding the deterioration of the recurrence rates over time after 6 and 8 years of follow-up, respectively [5, 50]. In the current investigation, we found that younger age at the time of surgery as well age at the time of initial instability was found to correlate negatively with early recurrence.

The current study has several limitations which can be summarized as follows: (1) The retrospective study design with all of the inherent issues associated with it. (2) No available pre-operative clinical scores for comparison. (3) No available radiographic data and the lack of information regarding the exact range of motion.

## Conclusion

Following the arthroscopic Bankart repair in 100 patients, the Kaplan-Meier failure-free survival estimates were 91% at 2 years and 70% at 10 years, which implies a significant drop over time. Failure rate was significantly affected by age at the time of surgery with 86% of recurrence cases observed in patients aged ≤30 years. This puts the effectiveness of the arthroscopic repair in this age group into question. Nevertheless, younger age at the time of surgery as well as at the time of initial instability was also associated with early recurrence within 2 years. Among those with recurrent instability, recurrence rate was found to be higher if there had been more than 5 instability episodes preoperatively. Return to the preinjury sport and occupational level was possible in 41 and 78%, respectively. These results have to be interpreted cautiously owing to the limited sample size.

## Data Availability

The datasets used and/or analyzed during the current study are available from the corresponding author on reasonable request.

## Abbreviations

ASES: American Shoulder and Elbow Surgeons Score
